# Downregulated formyl peptide receptor 2 expression in the epileptogenic foci of patients with focal cortical dysplasia type IIb and tuberous sclerosis complex

**DOI:** 10.1002/iid3.706

**Published:** 2022-10-26

**Authors:** Kaixuan Huang, Zhongke Wang, Zeng He, Yang Li, Shujing Li, Kaifeng Shen, Gang Zhu, Zhonghong Liu, Shengqing Lv, Chunqing Zhang, Hui Yang, Xiaolin Yang, Shiyong Liu

**Affiliations:** ^1^ Department of Neurosurgery, Epilepsy Research Center of PLA, Xinqiao Hospital Army Medical University (Third Military Medical University) Chongqing China; ^2^ Department of Neurosurgery Armed Police Hospital of Chongqing Chongqing China

**Keywords:** focal cortical dysplasia type IIb, formyl peptide receptor 2, inflammation resolution, resolvin D1, tuberous sclerosis complex

## Abstract

**Background:**

Focal cortical dysplasia type IIb (FCDIIb) and tuberous sclerosis complex (TSC) show persistent neuroinflammation, which promotes epileptogenesis and epilepsy progression, suggesting that endogenous resolution of inflammation is inadequate to relieve neuronal network hyperexcitability. To explore the potential roles of formyl peptide receptor 2 (FPR2), which is a key regulator of inflammation resolution, in epilepsy caused by FCDIIb and TSC, we examined the expression and cellular distribution of FPR2.

**Method:**

The expression of FPR2 and nuclear factor‐κB (NF‐κB) signaling pathway was examined by real‐time PCR, western blots, and analyzed via one‐way analysis of variance. The distribution of FPR2 was detected using immunostaining. The expression of resolvin D1 (RvD1, the endogenous ligand of FPR2) was observed via enzyme‐linked immunosorbent assay. Pearson's correlation test was used to evaluate the correlation between the expression levels of FPR2 and RvD1 and the clinical variants.

**Results:**

The expression of FPR2 was significantly lower in FCDIIb (*p* = .0146) and TSC (*p* = .0006) cortical lesions than in controls, as was the expression of RvD1 (FCDIIb: *p* = .00431; TSC: *p* = .0439). Weak FPR2 immunoreactivity was observed in dysmorphic neurons (DNs), balloon cells (BCs), and giant cells (GCs) in FCDIIb and TSC tissues. Moreover, FPR2 was mainly distributed in dysplastic neurons; it was sparse in microglia and nearly absent in astrocytes. The NF‐κB pathway was significantly activated in patients with FCDIIb and TSC, and the protein level of NF‐κB was negatively correlated with the protein level of FPR2 (FCDIIb: *p* = .00395; TSC: *p* = .0399). In addition, the protein level of FPR2 was negatively correlated with seizure frequency in FCDIIb (*p* = .0434) and TSC (*p* = .0351) patients.

**Conclusion:**

In summary, these results showed that the expression and specific distribution of FPR2 may be involved in epilepsy caused by FCDIIb and TSC, indicating that downregulation of FPR2 mediated the dysfunction of neuroinflammation resolution in FCDIIb and TSC.

## Graphical abstract

The expression of formyl peptide receptor 2 (FPR2) was significantly lower in focal cortical dysplasia type IIb (FCDIIb) and tuberous sclerosis complex (TSC) cortical lesions than in controls, and the protein level of FPR2 was negatively correlated with seizure frequency in FCDIIb and TSC patients, which indicate that FPR2 activation may be a potential strategy for attenuating seizures and for uniform progression of epilepsy in FCDIIb and TSC patients.

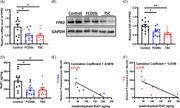

## 1 INTRODUCTION

Malformations of cortical development (MCDs) are caused by abnormal nerve cell proliferation, migration, and differentiation, which are important causes of refractory epilepsy.^1^ Focal cortical dysplasia type IIb (FCDIIb) and tuberous sclerosis complex (TSC) are two classic MCD phenotypes.^2^, ^3^ TSC is an inherited disease, while FCD is associated with somatic mutations. Thus, there are some differences between FCDIIb and TSC, such as TSC has microcalcification.^2^ However, both of them are involved in the mTOR signaling pathway and have similar histopathological features, such as lamination disorganization with dysmorphic neurons (DNs).^4^, ^5^ It has been confirmed that abnormal nerve cells and tissue structures are involved in epilepsy associated with FCDIIb and TSC.^6^ Furthermore, chronic persistent neuroinflammation has been observed in FCDIIb and TSC, which contributed to epilepsy.^7^ However, the neuroinflammatory mechanism in epilepsy associated with FCDIIb and TSC needs further elucidation.

We have shown that chronic neuroinflammation with the CD47 and CD200 signaling pathways was activated in FCD brain samples.^8^ Moreover, Toll‐like receptors (TLRs) and high‐mobility group box 1 (HMGB1) are upregulated in neurons and glial cells in FCDIIb and TSC.^9^, ^10^ HMGB1 belongs to the damage‐associated molecular patterns (DAMPs) and promotes the upregulation of downstream inflammatory factors in epilepsy, including nuclear factor‐κB (NF‐κB), interleukin‐1β (IL‐1β), and tumor necrosis factor‐α (TNF‐α).^11^ In addition, glucocorticoid therapy has achieved the prevention and treatment of epilepsy and epileptic encephalopathy by directly inhibiting the expression and release of IL‐1β and TNF‐α,^12^, ^13^ but has serious side effects (such as hypertension and osteoporosis).^14^ Intriguingly, a recent study suggests that inadequate pro‐resolving progress sustains neuroinflammation during epileptogenesis.^15^ These results suggest that active inflammation resolution, rather than inflammation suppression, may be a better option for epilepsy caused by FCDIIb and TSC.

Formyl peptide receptor 2 (FPR2) is the key factor in switching and balancing inflammation.^16^ FPR2 is a seven‐transmembrane G‐protein‐coupled receptor that interacts with various ligands performing different effects, such as DAMPs and serum amyloid A1‐stimulating proinflammation, resolvin D1 (RvD1), annexin A1 (ANXA1), and lipoxin A4 triggering the resolution of inflammation.^16^, ^17^, ^18^ However, FPR2 commonly participates in the active process of inflammation resolution in the central nervous system.^19^, ^20^, ^21^ FPR2 activated by ANXA1 downregulated IL‐6 activation in cerebral hemorrhage mice model.^22^ Moreover, FPR2 antagonizes lipopolysaccharide‐induced microglial activation mainly by inhibiting the NF‐κB pathway.^23^ FPR2 also decreases neuroinflammation by combining RvD1.^24^ RvD1 can restore and improve neurological function by strongly inhibiting neutrophil infiltration and microglial activation through FPR2.^25^ Furthermore, RvD1 was increased in the hippocampus in epilepsy mice model during acute seizures to protect neurons against epileptogenesis.^15^ Additionally, FPR2 also mediates the migration of neurons in vitro and in vivo and promotes the differentiation of neurons during cerebral development.^26^ These results indicated that FPR2 could have important roles in FCDIIb and TSC, which results from abnormal cortical developments.

Therefore, to explore the roles of FPR2 in cortical lesions of FCDIIb and TSC patients, we examined the expression and cellular distribution of FPR2 and its endogenous ligand RvD1 and the downstream NF‐κB signaling pathway. Furthermore, we analyzed the correlations between the expression levels of FPR2 and RvD1 and the clinical variables of patients.

## 2 MATERIALS AND METHODS

### 2.1 Subjects

Fifty‐two human cerebral cortex specimens were collected from the Neurosurgery Department of Xinqiao Hospital (Army Medical University, Chongqing, China) and were divided into three groups: the FCDIIb (*n* = 20), TSC (*n* = 20), and control groups (*n* = 12). All procedures for this study were conducted in accordance with guidelines approved by the Army Military Medical University Ethics Committee (ethical approval number: 2022‐361‐01). All specimens were collected and used according to the Declaration of Helsinki. Each patient underwent a complete preoperative evaluation and signed an informed consent form for research using the removed brain tissue. Each case was diagnosed by two neuropathologists according to the International League Against Epilepsy (ILAE) classification.^27^ The seizure types were classified based on the latest classification system of the ILAE. Control tissue was collected from autopsies of 12 patients who had no history of epilepsy or any other neurological diseases. Referring to the methods in our previous research,^8^ all tissues were collected no more than 24 h after death because most proteins are stable in this time interval.^28^

### 2.2 Tissue preparation

During surgery, all cortical lesions were divided into two tissue blocks. One block was fixed in 10% buffered formalin for 3 days and then embedded with paraffin. The thickness of paraffin‐embedded sections was 5 µm and was used for histopathologic diagnosis and pathologic classification, followed by immunohistochemical and immunofluorescence staining. Another block was soaked in a solution of buffered diethylpyrocarbonate (1:1000) for 1 day and immediately frozen in liquid nitrogen. The frozen tissues were stored at −80°C until being used for enzyme‐linked immunosorbent assay (ELISA), quantitative real‐time polymerase chain reaction (real‐time PCR), and western blot analysis.

### 2.3 Real‐time PCR

Total RNA was isolated from the FCDIIb, TSC, and control specimens by using a TRIzol RNA separation reagent (Invitrogen). PCR primers were designed according to the principle of DNA complementary sequences. The Real‐Time PCR protocol included 1 μl of complementary DNA, 7 μl of SYBR Green mix (TaKaRa), 1.6μl of ddH_2_O, and 0.2 μl of forward and reverse primers. The in vitro amplification conditions were as follows: 1 cycle of 95°C for 5 min, followed by 39 cycles of 95°C for 30 s and 68°C for 30 s. Finally, the quantitative results were calculated by the $2^{-\Delta \Delta C_{T}}$ method. All primers used are shown in Supporting Information: Table 1.

### 2.4 Western blots

Total protein was extracted from human tissues by using RIPA Lysis Buffer (Beyotime). The protein concentration was determined by a BCA Protein Assay Kit (Bio‐Rad). Sodium dodecyl sulfate‐polyacrylamide gel electrophoresis was used, and equal amounts of protein (20 µg) were added to each electrophoresis well on the gel plate. Next, a semidry electroblotting system (Bio‐Rad) was used to transfer the electrophoretic proteins onto a polyvinylidene fluoride membrane (Millipore). The blots were incubated in Tris‐buffered saline with Tween (TBST, 20 mmol/L Tris‐HCl, pH 8.0, 150 mmol/L NaCl, 0.5% Tween‐20) and 5% skim milk for 2 h at 37°C. Then, the blots were incubated for 12 h at 4°C in TBST containing primary antibodies against FPR2 (1:800; Abcam), NF‐κB (1:500; Cell Signaling Technology), and GAPDH (1:1000; Cell Signaling Technology). After the blots were washed in TBST, they were incubated with horseradish peroxidase‐conjugated secondary antibodies (1:1000; Zhongshan Goldenbridge) for 2 h at 37°C. The protein bands on the membranes were observed with enhanced chemiluminescence, and densitometry was calculated using ImageJ software (National Institutes of Health). GAPDH levels were evaluated as a loading control.

### 2.5 Immunohistochemistry and immunofluorescence analysis

Paraffin‐embedded slices (5 µm) were used for immunohistochemistry and immunofluorescence. First, the sections were incubated with an anti‐FPR2 (1:500; Abcam) primary antibody overnight at room temperature. Second, these sections were incubated with secondary antibodies conjugated to a peroxidase‐labeled dextran polymer (Envision + System‐HRP; Boster) for 1 h at 37°C. Finally, these sections were stained with hematoxylin, dehydrated, and coverslipped. For double‐labeling immunofluorescence staining, sections were incubated at 4°C for 12 h with the following antibodies: anti‐FPR2 (1:500; Abcam), anti‐NF200 (1:500; Millipore), anti‐glial fibrillary acidic protein (GFAP) (1:500; Sigma), and anti‐HLA‐DR (1:500; Abcam). These sections were then incubated with the corresponding Cy3‐ and 488‐conjugated secondary antibodies (both at 1:500; Invitrogen) for 2 h at 37°C. Next, these sections were incubated with 4′,6‐diamidino‐2‐phenylindole (Beyotime) for 3 min at room temperature. Finally, the fluorescent sections were imaged with a Zeiss Axio Vert microscope (Zeiss).

### 2.6 Evaluation of immunostaining

Briefly, the stained sections were evaluated using a light microscope (BX51; Olympus) according to the previous protocol.^29^ The intensity of FPR2 staining was assessed by a three‐point scale (0: −, no; 1: ±, weak; 2: +, moderate; 3: ++, strong immunoreactivity [IR]). The relative number of FPR2‐positive cells within the sections of patients was assessed by the frequency scores (i, less than 10%; ii, 11%–50%; iii, >50%). The product of the intensity and frequency score was calculated as the IR score. In addition, the mean optical density (MOD) of FPR2 was calculated using the same procedure as given in a previous study.^30^ The photographs and MOD were analyzed with Image‐Pro Plus software (Media Cybernetics).

### 2.7 ELISA

Frozen cortical samples were collected and cleaned in precooled phosphate‐buffered saline to remove the blood. Then, the samples were thoroughly ground, the homogenate was centrifuged at 5000 *g* for 5min, and the supernatant was reserved for subsequent detection with an RvD1 ELISA kit (Cayman Chemicals). The plate was cleaned to remove any unbound reagents, and Ellman's reagents were added to each well. After incubation overnight at 4°C, the product of this enzymatic reaction had a distinct yellow color and the optical density was measured at 420 nm with a plate reader (Bio‐Rad). The concentrations of the samples were calculated on the basis of the standard curve.

### 2.8 Statistical analysis

Differences in age, sex, seizure frequency, and epilepsy duration were evaluated using *t* tests. Pearson's correlation test was used to perform bivariate correlation analyses. Statistical analysis between groups was used for one‐way analysis of variance, followed by Tukey's test. A *p* < .05 indicated a significant difference. The data are shown as the mean ± SEM, and the analyses were performed using GraphPad Prism 8.4.2 (GraphPad Software Inc.).

## 3 RESULTS

### 3.1 Clinical characteristics

The study included 20 FCDIIb, 20 TSC, and 12 control cortex samples. The average age of the control subjects was 12.54 ± 1.843 years, with 7 females and 5 males; the average age of the FCDIIb patients was 8.36 ± 0.921 years, with 10 females and 10 males; and the average age of the TSC patients was 10.0 ± 1.083 years, with 12 females and 8 males. There were no significant differences in sex or age between the control subjects and the FCDIIb and TSC patients (sex: *p* > .05; age: *p* > .05). Detailed data for the controls and FCDIIb and TSC patients are shown in Supporting Information: Tables 2 and 3.

### 3.2 Expression of FPR2 and RvD1 in specimens from FCDIIb and TSC patients

**Figure 1:**
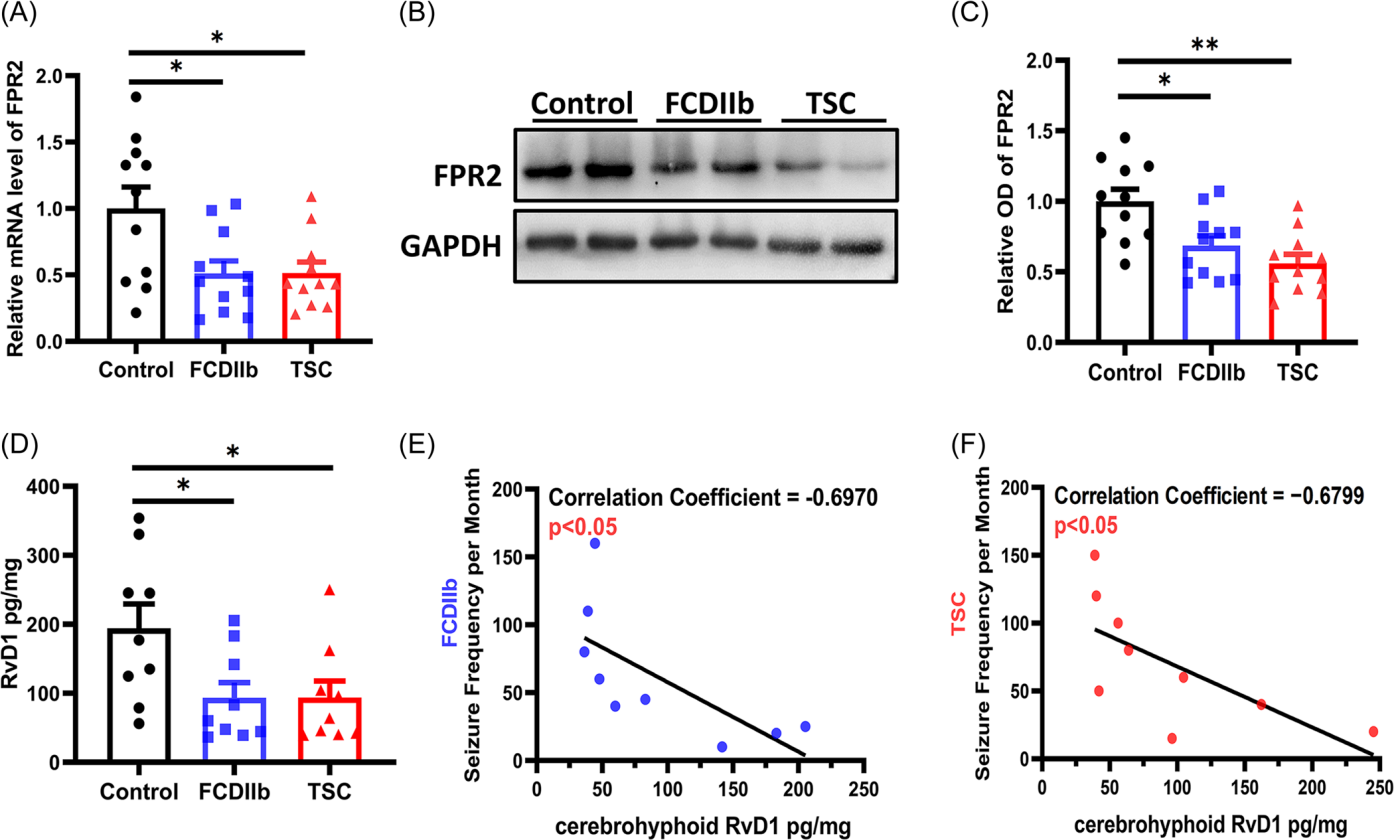
Expression of FPR2 and RvD1 in FCDIIb and TSC patients and controls. (A) FPR2 mRNA expression levels were examined by RT‐PCR. FPR2 mRNA levels were significantly decreased in the FCDIIb (*n* = 11, *p = .0182) and TSC (*n* = 11, *p = .0188) samples compared to the control samples. (B, C) Representative western blots and densitometric analysis showing the FPR2 protein levels. FPR2 expression levels were significantly decreased in FCDIIb (*n* = 11, *p = .0146) and TSC (*n* = 11, **p = .0006) samples compared to control samples. (D) Quantification of cerebral tissue RvD1 levels as determined by ELISA. Compared with the control group, RvD1 was downregulated in the FCDIIb (*n* = 9, *p = .0431) and TSC (*n* = 9, *p = .0439) groups. (E, F) Correlations between the expression of RvD1 and seizure frequency in FCDIIb and TSC patients. There were significant negative correlations in FCDIIb (*n* = 11, *r* = −0.6970, *p = .0369) and TSC (*n* = 11, *r* = −0.6799, *p = .0439) patients, as shown in the scatter plot. All data are shown as the mean ± SEM. ELISA, enzyme‐linked immunosorbent assay; FCDIIb, focal cortical dysplasia type IIb; FPR2, formyl peptide receptor 2; mRNA, messenger RNA; RvD1, resolvin D1; RT‐PCR, reverse transcription‐polymerase chain reaction; TSC, tuberous sclerosis complex.

Real‐time PCR was used to detect the messenger RNA (mRNA) levels of FPR2 in surgical samples from the FCDIIb, TSC, and control groups. The results showed that the mRNA levels of FPR2 in the FCDIIb and TSC groups were significantly decreased compared with those in the control group (Figure 1A; FCDIIb: *p* = .0182; TSC: *p* = .0188).

To observe whether the protein levels were also downregulated in the cortical foci of FCDIIb and TSC, the expression levels of FPR2 were examined by western blot analysis. The expression of FPR2 in the FCDIIb and TSC groups was significantly decreased compared with that in the control group (Figure 1B,C; FCDIIb: *p* = .0146; TSC: *p* = .0006). Furthermore, ELISA was used to measure the expression levels of RvD1 (the endogenous ligand of FPR2) in the specimens of patients with FCDIIb or TSC and controls. This result revealed that the expression of RvD1 in the FCDIIb and TSC samples was significantly decreased compared to that in the control samples (Figure 1D; FCDIIb: *p* = .0431; TSC: *p* = .0439).

Next, correlations between the expression level of RvD1 and different clinical variables were analyzed by Pearson's correlation test. The expression of RvD1 in FCDIIb and TSC patients was negatively correlated with seizure frequency (Figure 1E,F; FCDIIb: *r* = −0.6790, *p* = .0369; TSC: *r* = −0.6799, *p* = .0439). These results indicate that FPR2 and RvD1 might be involved in epilepsy caused by FCDIIb and TSC.

### 3.3 Immunohistochemical properties of FPR2 in patients with FCDIIb and TSC

**Figure 2:**
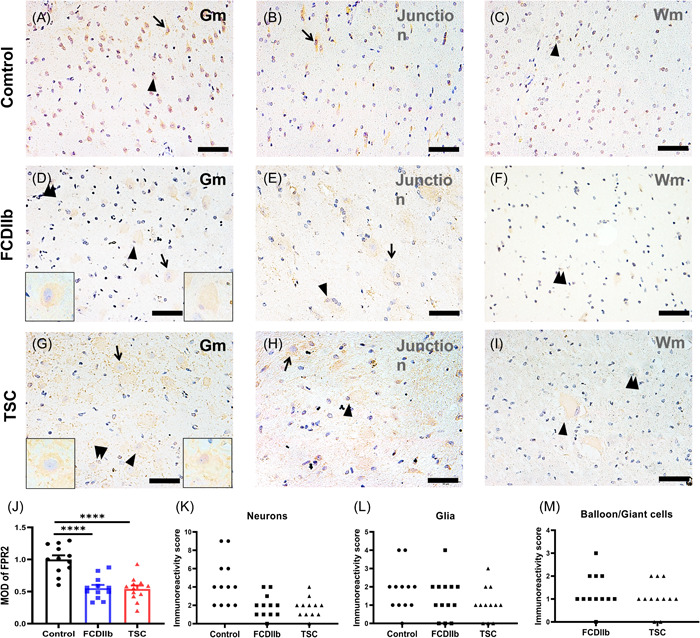
Distribution of FPR2 IR in FCDIIb and TSC patients and controls. (A, C) Representative micrographs of FPR2 IR in control gray matter (A, Gm), gray/white matter junction (B, Junction), and white matter (C, Wm) tissue showing moderate FPR2 IR in neurons (arrows) and in glia (arrowheads). (D–F) Representative photos of FPR2 IR in FCDIIb gray matter (D, Gm), gray/white matter junction (E, Junction), and white matter tissue (F, Wm). (D) DNs (arrows, left inset) and BCs (arrowheads, right inset) showed weak FPR2 IR in FCDIIb specimens and weak FPR2 IR in glial cells (double arrowheads). (G–I) Representative micrographs of FPR2 IR staining in TSC gray matter (G, Gm), gray/white matter junction (H, Junction), and white matter (I, Wm) tissues. (G) DNs (arrows, left inset) and GCs (arrowheads, right inset) showed weak FPR2 IR in TSC specimens and weak FPR2 IR in glial cells (double arrowheads). (J) The MOD of FPR2 in the FCDIIb (*n* = 12, ****p < .0001) and TSC (*n* = 12, ****p < .0001) groups was decreased compared to that in the control group. (K–L) Distribution of FPR2 in the control, FCDIIb and TSC groups in neurons (K), glia (L), and BCs/GCs (M). The IR score is the product of the intensity score and frequency score (details for Materials and methods section). Scale bar = 50 µm. All data are shown as the mean ± SEM. BC, balloon cell; DN, dysmorphic neurons; FCDIIb, focal cortical dysplasia type IIb; FPR2, formyl peptide receptor 2; IR, immunoreactivity; MOD, mean optical density; TSC, tuberous sclerosis complex.

**Figure 3:**
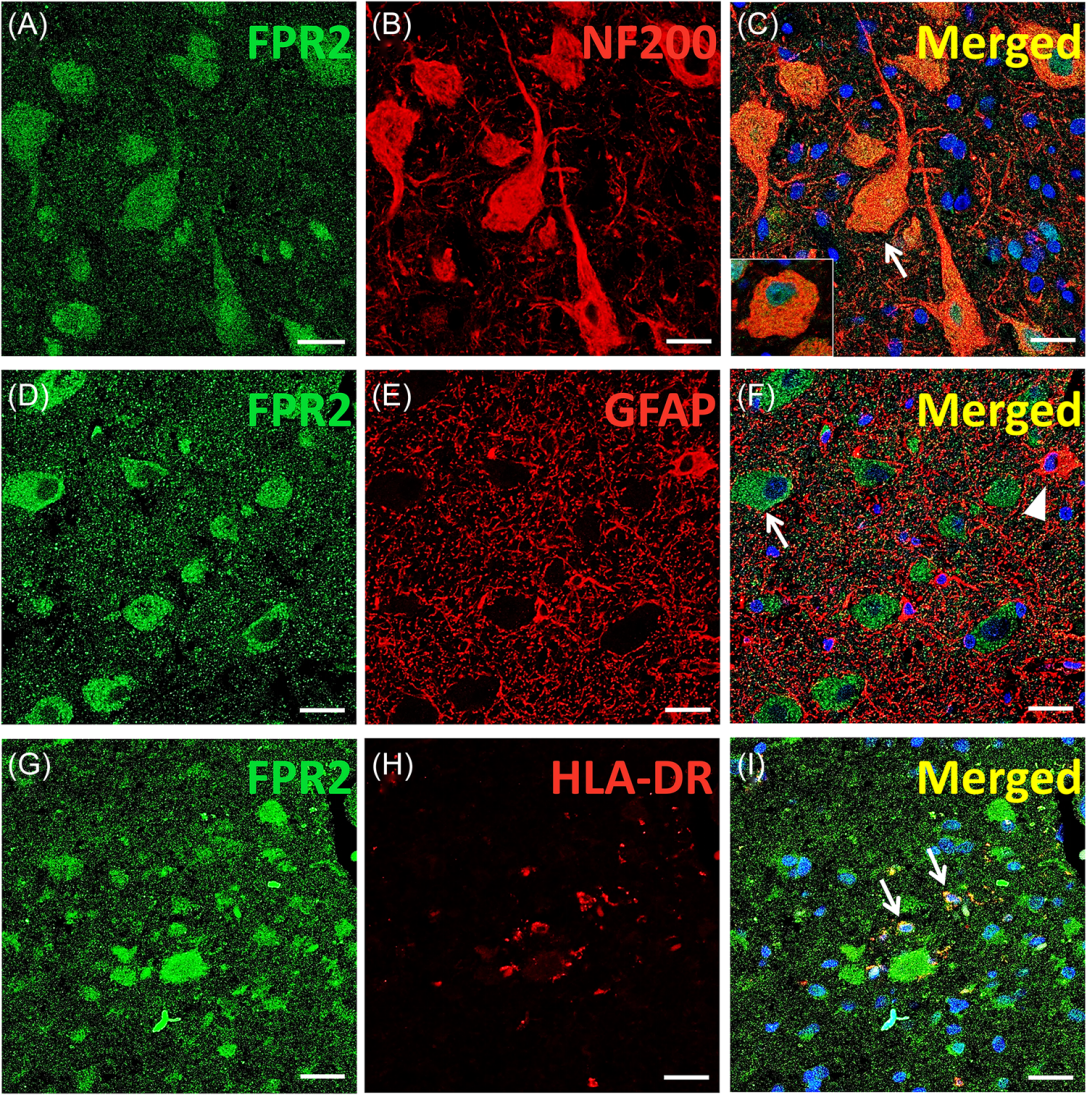
Immunofluorescence of FPR2 in FCDIIb tissues. (A–C) Double labeling showed colocalization of FPR2 (A, green) with NF200 (B, red), and FPR2 was expressed in NF200‐positive DNs (C, arrow) and BCs (C, inset). (D–F) Double labeling did not show colocalization of FPR2 (D, green) with GFAP (E, red). The arrow and arrowhead in (F) indicate the FPR2‐positive DNs and GFAP‐positive astrocytes, respectively. (G–I) Double labeling showed colocalization of FPR2 (G, green) with HLA‐DR (H, red) in microglia (I, arrows). Scale bar = 20 µm. BC, balloon cell; DN, dysmorphic neurons; FCDIIb, focal cortical dysplasia type IIb; FPR2, formyl peptide receptor 2; GFAP, glial fibrillary acidic protein; MOD, mean optical density; TSC, tuberous sclerosis complex.

**Figure 4:**
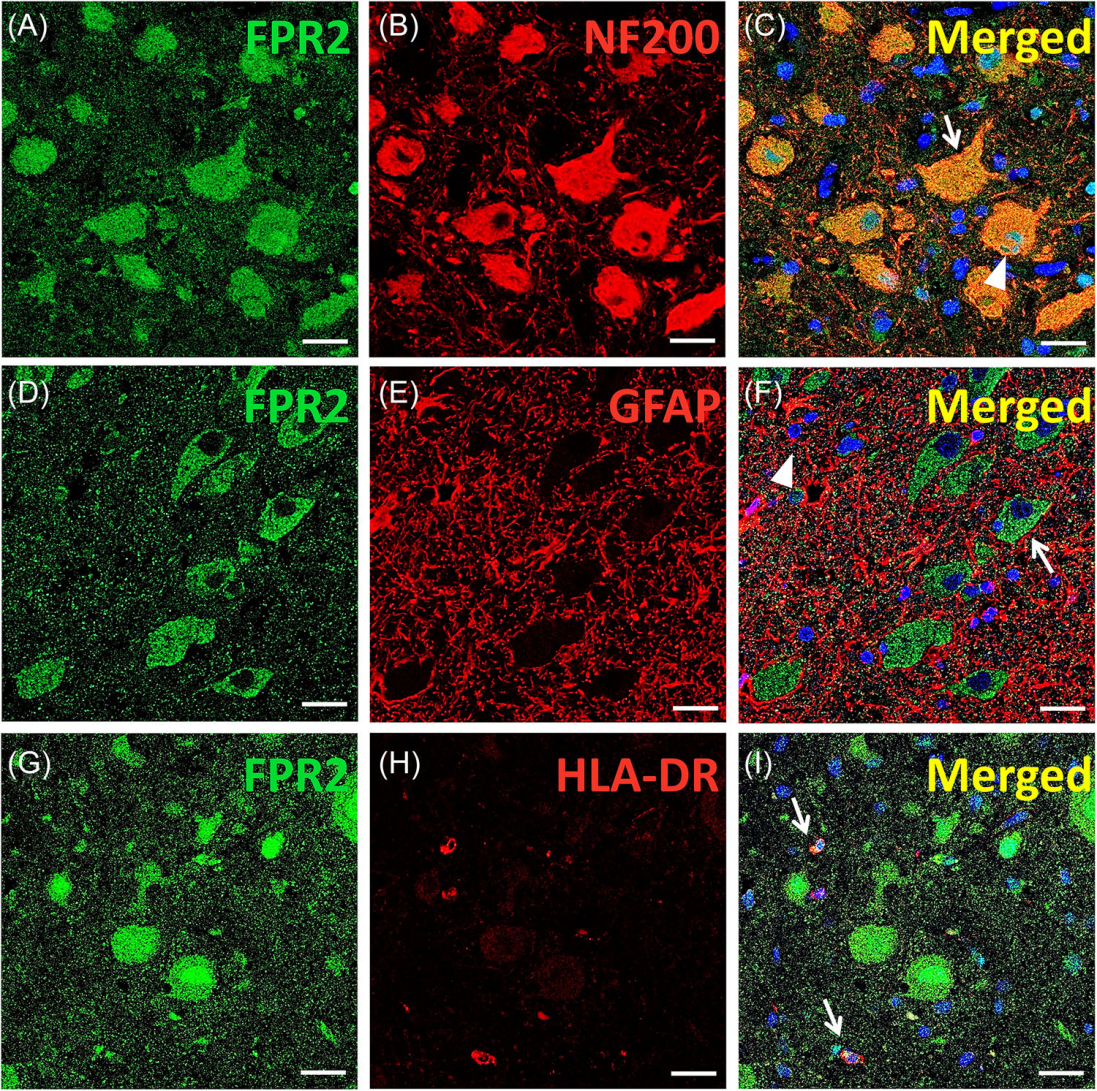
Immunofluorescence of FPR2 in TSC tissues. (A–C) Double labeling showed colocalization of FPR2 (A, green) with NF200 (B, red) in DNs (C, arrow) and GCs (C, arrowhead). (D–F) Double labeling showed colocalization of FPR2 (D, green) with GFAP (E, red) but not with GFAP‐positive astrocytes (F, arrowhead). (G–I) Double labeling showed that FPR2 (G, green) and HLA‐DR (H, red) were coexpressed in microglia (I, arrows). Scale bar = 20 µm. BC, balloon cell; DN, dysmorphic neurons; FCDIIb, focal cortical dysplasia type IIb; FPR2, formyl peptide receptor 2; GFAP, glial fibrillary acidic protein; TSC, tuberous sclerosis complex.

To further understand FPR2, we determined the IR and cellular distribution of FPR2 in specimens using immunohistochemistry and immunofluorescence. The results showed moderate FPR2 IR in the neurons and moderate to weak IR in glial cells in gray matter, white matter, and junctions of gray/white matter in controls. Weak FPR2 IR was observed in 74.68 ± 3.44% of DNs (*n*= 321) and 89.44 ± 3.25% of balloon cells (BCs) (*n* = 98) in the FCDIIb group (Figure 2D). In the TSC group (Figure 2G), weak FPR2 IR was also observed in 79.14 ± 2.73% of DNs (*n* = 363) and 85.18 ± 4.26% of giant cells (GCs) (*n* = 112). Moreover, weak IR was observed in glial cells in patients with FCDIIb and TSC. Moreover, the MOD of FPR2 was lower in both FCDIIb and TSC patients than in controls (Figure 2J; FCDIIb: *p* < .0001; TSC: *p* < .0001). In addition, double‐labeling immunofluorescence of the FCDIIb group showed that FPR2 was expressed in NF200‐positive DNs (Figure 3A); FPR2 and HLA‐DR were coexpressed in microglia (Figure 3C), but FPR2 was not expressed in GFAP‐positive astrocytes (Figure 3B). Double‐labeling immunofluorescence of the TSC group also showed that FPR2 was expressed in NF200‐positive DNs (Figure 4D) and in HLA‐DR‐positive microglia (Figure 4F), but FPR2 was not expressed in GFAP‐positive astrocytes (Figure 4E). The IR and specific distribution of FPR2 suggest that FPR2 can mainly regulate the activity of dysplastic neurons and some microglia in FCDIIb and TSC.

### 3.4 NF‐κB pathway in patients with FCDIIb and TSC

**Figure 5:**
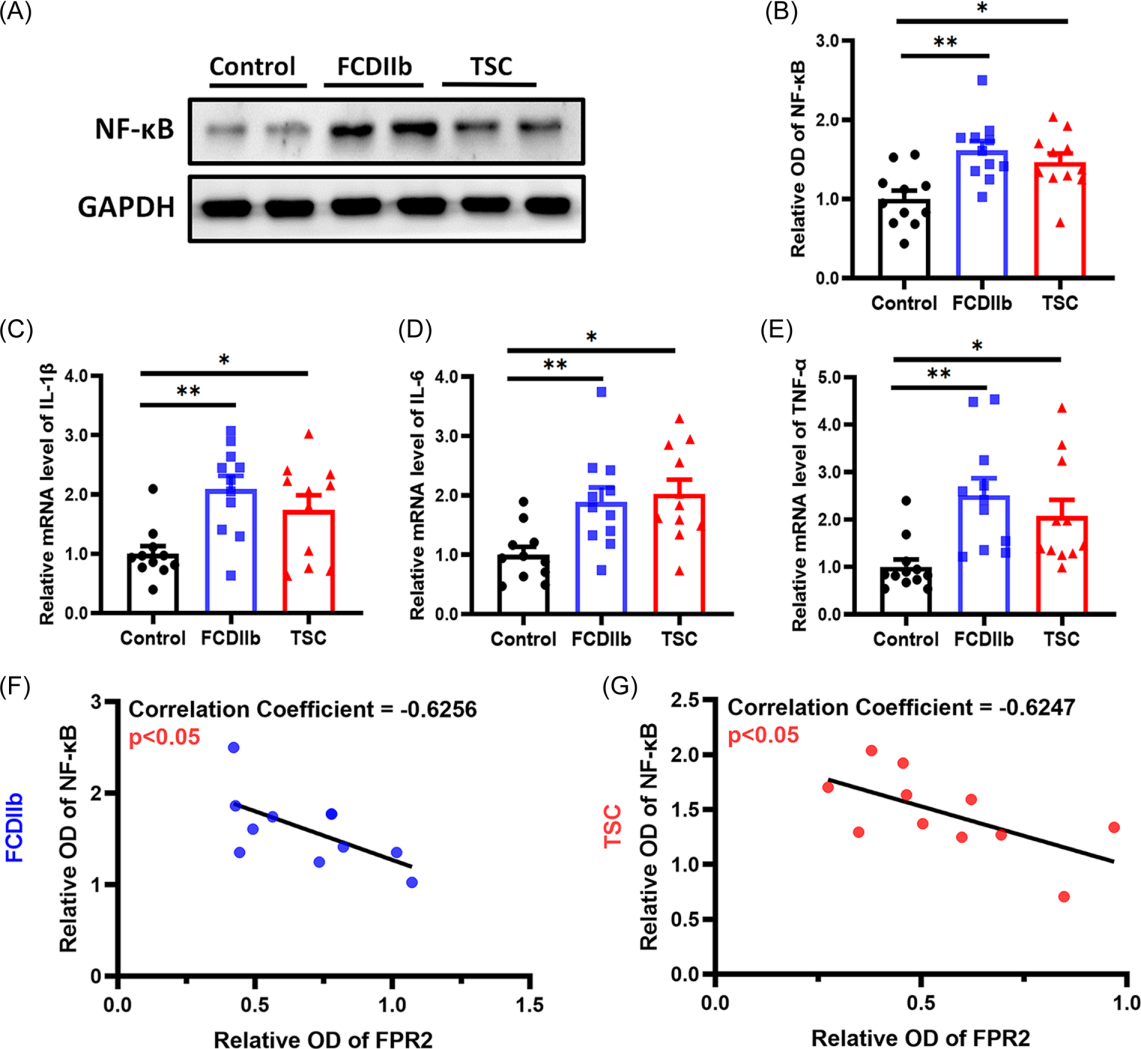
Activation of inflammation in FCDIIb and TSC patients. (A) Representative western blots showing NF‐κB expression in surgical samples. (B) The protein levels of NF‐κB in FCDIIb (*n* = 11, **p = .0015) and TSC (*n* = 11, *p = .0165) patients were higher than those in controls. (C–E) RT–PCR results showed that the mRNA levels of IL‐β (C), IL‐6 (D) and TNF‐α (E) were significantly higher in FCDIIb (*n* = 11; IL‐β: **p = .0021, IL‐6: *p = .0152, TNF‐α: **p = .0025) and TSC (*n* = 11; IL‐β: *p = .0419, IL‐6: **p = .0050, TNF‐α: *p = .0348) samples than in control specimens. (F, G) Correlations between NF‐κB and FPR2 expression levels in the FCDIIb and TSC groups. There were significant negative correlations in FCDIIb (*n* = 11, *r* = −0.6256, *p = .0395) and TSC (*n* = 11, *r* = −0.6247, *p = .0399) patients, as shown in the scatter plot. All data are shown as the mean ± SEM. FCDIIb, focal cortical dysplasia type IIb; FPR2, formyl peptide receptor 2;  GAPDH, glyceraldehyde 3‐phosphate dehydrogenase; NF‐κB, nuclear factor‐κB; RT‐PCR, reverse transcription‐polymerase chain reaction; TNF‐α, tumor necrosis factor‐α; TSC, tuberous sclerosis complex.

The above results suggest that FPR2 downregulation is involved in epilepsy caused by FCDIIb and TSC. Moreover, FPR2 has been reported to ameliorate neuroinflammation through the inflammation signaling pathway.^19^ Next, we explored the NF‐κB inflammatory pathway, which has been proven to play important roles in epileptogenesis in FCDIIb and TSC. The NF‐κB pathway was significantly activated in the FCDIIb and TSC groups (Figure 5A,B; FCDIIb: *p* = .0015; TSC: *p* = .0165). The mRNA expression levels of the inflammatory factors IL‐1β (Figure 5C; FCDIIb: *p* = .0021, TSC: *p* = .0419), IL‐6 (Figure 5D; FCDIIb: *p* = .0152, TSC: *p* = .0050), and TNF‐α (Figure 5E; FCDIIb: *p* = .0025, TSC: *p* = .0348) were also increased in the FCDIIb and TSC groups. To explore the relationship between FPR2 and NF‐κB, we analyzed the correlation between NF‐κB and FPR2 protein levels. NF‐κB expression was negatively associated with FPR2 levels in the FCDIIb and TSC samples from patients (Figure 5F,G; FCDIIb: *r* = −0.6256, *p* = .0395; TSC: *r* = −0.6247, *p* = .0399). These results indicate that FPR2 can regulate neuroinflammation in FCDIIb and TSC, but more studies are needed to confirm this hypothesis.

### 3.5 Correlation between the protein levels of FPR2 and clinical features in FCDIIb and TSC patients

**Figure 6:**
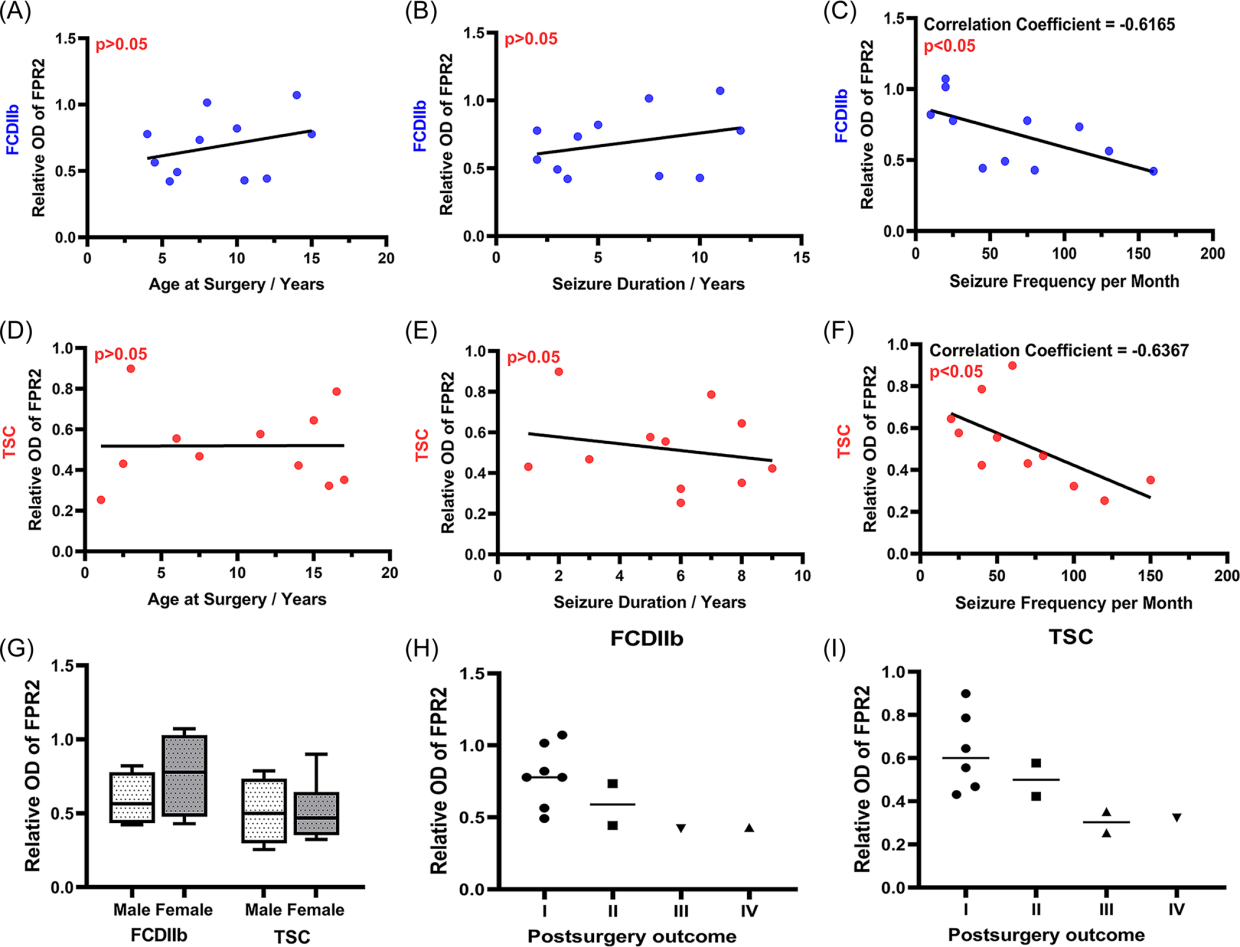
Correlations between the protein levels of FPR2 and various clinical features in FCDIIb and TSC patients. (A–I) There were significant negative correlations between FPR2 expression levels and seizure frequency in the FCDIIb (C, *n* = 11, r = −0.6165, *p* = .0434) and TSC (F, *n* = 11, *r* = −0.6367, *p = .0351) groups but not between FPR2 expression levels and other clinical variables, as shown in the scatter plot. FCDIIb, focal cortical dysplasia type IIb; FPR2, formyl peptide receptor 2; OD, optical density; TSC, tuberous sclerosis complex.

To further explore the relationship between FPR2 and epilepsy in FCDIIb and TSC patients, the correlations between FPR2 protein expression levels and various clinical variables (age, sex, postsurgery outcome, age at onset, age at surgery, seizure frequency per month, duration of seizures, duration of epilepsy) were analyzed by univariate analysis. Notably, there were significant negative correlations between FPR2 expression levels and seizure frequency in FCDIIb patients (Figure 6C; *r* = −0.6165, *p* = .0434) and TSC patients (Figure 6F; *r* = −0.6367, *p* = .0351), but the obvious correlations were not observed for the other clinical variables.

## 4 DISCUSSION

In this study, we found that the expression of FPR2 and its endogenous ligand RvD1 was significantly decreased in cortical lesions in FCDIIb and TSC patients and negatively correlated with seizure frequency. FPR2 expression was mainly reduced in dysplastic neurons in FCDIIb and TSC. Double‐labeling immunofluorescence indicated that FPR2 was mainly expressed in dysplastic neurons and sparsely expressed in microglia but not in astrocytes. Moreover, NF‐κB and its downstream factors including IL‐1β, IL‐6, and TNF‐α were upregulated in FCDIIb and TSC. Furthermore, the NF‐κB expression levels were negatively related to FPR2 levels in FCDIIb and TSC patients. These results suggested that the decrease in FPR2 levels was involved in epilepsy in FCDIIb and TSC patients.

Increasing evidence suggests that inflammation enhances neuronal excitability to promote seizures and epileptogenesis in epileptic patients and animals.^31^ Notably, inflammatory markers are demonstrated in fetal brains in TSC as early as midgestation in the human.^32^ Although FCDIIb and TSC are different in gene mutation and microstructure, the pathogenesis of epilepsy caused by neuroinflammation involved in innate immunity is similar.^33^, ^34^ Moreover, a wide range of inflammatory factors, such as HMGB1, TLR4, IL‐1β, TNF‐α, and IL‐6, are upregulated in FCD and TSC.^29^ HMGB1‐TLR4 further activate the NF‐κB signaling pathway through the TRIF (Toll/IL‐1R domain‐containing adapter‐inducing interferon‐β) in epileptic focus.^11^ In addition, a recent study reports that the inadequate pro‐resolving progress sustains the above neuroinflammation during epileptogenesis.^15^ FPR2, as a key regulator of inflammation resolution, can be activated by various endogenous ligands, thus performing different functions including proinflammatory roles and inflammation resolution.^19^, ^35^ Intriguingly, FPR2 often promote neuroinflammation resolution in the central nervous system.^19^, ^20^, ^21^ FPR2 activation attenuates neuroinflammatory responses in subarachnoid rats.^36^ Furthermore, FPR2 knockout mice present an exaggerated cerebral inflammatory response after ischemia/reperfusion injury.^37^ In this study, FPR2 expression was significantly decreased in FCDIIb and TSC patients; moreover, FPR2 expression levels were negatively correlated with preoperative seizure frequency in FCDIIb and TSC patients, suggesting that FPR2 downregulation could result in the inadequate inflammation resolution that contributes to epilepsy in FCDIIb and TSC patients. But the underlying mechanism is obscure.

RvD1, as an endogenous high‐affinity ligand of FPR2, is synthesized by continuous oxidation of DHA by 5‐lipoxygenase (LOX) and 15‐LOX and has been elucidated to trigger active inflammation resolution in vivo and in vitro.^38^ A recent study also shows that RvD1 promotes an inflammatory resolution to control seizures in the acute phase of the epileptic mice model.^15^ Moreover, RvD1 reduces neuroinflammation by activating FPR2 after hypoxic–ischemic injury in neonatal rats.^39^ Furthermore, the RvD1–FPR2 interaction promotes neuroinflammation resolution by inhibiting microglial proinflammatory polarization and regulating microglial switching to the anti‐inflammatory M2 phenotype.^40^, ^41^ Lipopolysaccharide (LPS)‐induced upregulation of IL‐1β and TNF‐α in microglia is significantly restricted by FPR2 activation.^23^ Besides, RvD1 alleviates inflammation in trophoblasts caused by chorioamnionitis in vivo and in vitro through the FPR2/NF‐κB pathway.^42^ In this study, the expression of FPR2 and RvD1 was both downregulated and negatively correlated with the preoperative seizure frequency of FCDIIb and TSC patients, and the expression of FPR2 was decreased in microglia and absent in astrocytes. Similarly, a recent study found that FPR2 was not expressed in astrocytes.^40^ In addition, the protein levels of NF‐κB were negatively correlated with the levels of FPR2 in FCDIIb and TSC. These results suggested that RvD1–FPR2 might be involved in the inadequate inflammation resolution in FCDIIb and TSC through the NF‐κB pathway of microglia, but not astrocytes, to contribute to the hyperexcitability of neurons and networks; however, more studies are needed to elucidate this possibility.

FPR2 might directly regulate the neuronal excitability. RvD1/FPR2 also inhibits the neuronal excitability by reducing the TRPV1 current.^43^ Similarly, AC2‐26 relieves inflammatory pain by desensitization of TRPV1 at the dorsal root ganglion neuron level through the FPR2–PLCβ‐Ca^2+^–CAM signaling pathway.^44^ Moreover, inhibiting the FPR2 signaling pathway significantly reduces the axonal growth of rat hippocampal neurons.^45^ In this study, FPR2 expression was mainly reduced in DNs, which are the sources of neuronal hyperexcitability in FCDIIb and TSC.^46^ We also observed that FPR2 was downregulated in BCs and GCs of FCDIIb and TSC. Moreover, BCs and GCs are considered to be involved in neuroinflammation in FCDIIb and TSC.^29^ Furthermore, FPR2 IR was sparsely observed in glial cells in FCDIIb and TSC. These results suggest that FPR2 might regulate dysplastic neuronal excitability and proinflammatory effects in FCDIIb and TSC.

Previous studies have shown that DHA participates in the development of the brain.^47^ Mutations in the gene encoding the DHA transporter (MFSD2A) cause lethal microcephaly syndrome, which is a subtype of MCD.^48^ Moreover, clinical treatment with DHA reduces seizure frequency in epilepsy patients.^49^ RvD1 is derived from the biosynthesis of DHA through 5‐LOX and 15‐LOX. RvD1 increases the expression of brain‐derived neurotrophic factor in brain neurons, which is closely related to brain development.^50^ Besides, FPR2 is also expressed in neural stem cells (NSCs) during cerebral development and mediates the migration of neurons in vitro and in vivo, in addition to promoting the differentiation of neurons into neurons.^26^ Although the relationship among RvD1, FPR2, and MCDs is still unclear. We observed that the expression levels of both RvD1 and FPR2 were decreased in cortical lesions of FCDIIb and TSC and that FPR2 was mainly expressed in DNs, BCs, and GCs, which partly have NSC properties.^51^ These results indicate that DHA might be involved in brain development through the RvD1–FPR2 signaling pathway. However, it is unclear whether MCDs caused decreases in RvD1 and FPR2 expressions or whether low RvD1 and FPR2 expressions were caused by the MCDs. However, this issue needs further study.

## 5 CONCLUSION

Collectively, FPR2 and RvD1 expressions are involved in epilepsy in FCDIIb and TSC patients. The decreases in RvD1–FPR2 might contribute to unresolved neuroinflammation via the NF‐κB pathway in FCDIIb and TSC. The results indicate that FPR2 activation may be a potential strategy for attenuating seizures and for uniform progression of epilepsy in FCDIIb and TSC patients.

## 6 LIMITS

Previous studies demonstrate that total RNA unchanged for up to 36 h postmortem.^28^, ^52^ Although RNA slightly degraded, the protein levels remained stable, and the immunostaining profiles of most proteins remained unchanged even after postmortem interval of over 50 h.^53^, ^54^ Thus, the downregulation of FPR2 in FCDIIb and TSC patients is reliable compared to autopsy controls. However, since the limitations regarding the use of human samples in functional studies and the lack of an MCD model that is similar to human MCDs with epilepsy, the mechanism of RvD1–FPR2 signaling in FCDIIb and TSC has not been further demonstrated. Moreover, it is unclear whether MCDs or epilepsy caused decreases in RvD1 and FPR2 expression or whether low RvD1 and FPR2 expression caused MCDs with epilepsy. Meanwhile, due to the diversity of FPR2 effects, FPR2 knockout in animals cannot fully explain its effect on neuroinflammation resolution. The detailed cellular function and mechanism of FPR2 in epilepsy and the development of FCD and TSC need to be further researched to explore possible new directions for the control of MCDs and epilepsy caused by MCDs.

## AUTHOR CONTRIBUTIONS

*Experimental design*: Shiyong Liu, Xiaolin Yang, Shengqing Lv, Chunqing Zhang, and Hui Yang. *Experiment implementation*: Kaixuan Huang, Zhonghong Liu, Zeng He, Yang Li, Shujing Li, Kaifeng Shen, and Gang Zhu. *Analysis data and drafting of manuscript*: Kaixuan Huang, Zhongke Wang, and Xiaolin Yang.

## CONFLICT OF INTEREST

The authors declare no conflict of interest.

## Supporting information

Supporting information.

## Data Availability

Data are available on request due to privacy/ethical restrictions.
