# Supplementary material for: Downregulated formyl peptide receptor 2 expression in the epileptogenic foci of patients with focal cortical dysplasia type IIb and tuberous sclerosis complex
Source: Immun Inflamm Dis. 2022 Oct 26;10(11):e706. doi: 10.1002/iid3.706 (PMC9597500; doi:10.1002/iid3.706)
Supplement: Supplementary file 1 — Supporting information. [file IID3-10-e706-s001.docx]

| Primers Forward Reverse |
| --- |
| Human  FPR2 5′-TCTTGCTCTAGTCCTTACCTTGC-3′ 5′-AATGACAAACCGGATAATCCCTC-3′    IL-1β 5′-ATGATGGCTTATTACAGTGGCAA-3′ 5′-GTCGGAGATTCGTAGCTGGA-3′    IL-6 5′-ACTCACCTCTTCAGAACGAATTG-3′ 5′-CCATCTTTGGAAGGTTCAGGTTG-3′    TNF-α 5′-CCTCTCTCTAATCAGCCCTCTG-3′ 5′-GAGGACCTGGGAGTAGATGAG-3′    GAPDH 5′-GGAGTCCACTGGCGTCTTCA-3′ 5′-GTCATGAGTCCTTCCACGATACC-3′  Rat  IL1-β 5′-CGTGGGATGATGACGACCTG-3′ 5′-GCCACAGGCGATTTTGTCGTT-3′    IL-6 5′-CAGAGGATACCACCCACAACAGA-3′ 5′-GAACTCCAGAAGACCAGAGCAGA-3′    TNF-α 5′-ATCGGTCCCAACAAGGAGGA-3′ 5′-CGCTTGGTGGTTTGCTACG-3′    GAPDH 5′-GAAGGTCGGTGTGAACGGAT-3′ 5′-CCCATTTGATGTTAGCGGGAT-3′ |

**Supplementary Table 1**

Summary of the primers used in this study.

**Supplementary Table 2**

| Clinical characteristics of individual patients with FCDIIb and TSC | | | | | | | | | |  |
| --- | --- | --- | --- | --- | --- | --- | --- | --- | --- | --- |
| Patient No. | Sex | Pathologic Diagnosis | Age at Surgery, Years | Seizure Duration, Years | Seizure Type | Brain Region | Seizure Frequency per Month | Postsurgery outcome | Application Used |  |
| 1 | F | FCDIIb | 1.5 | 0.5 | GES | O | 40 | I | Elisa, IHC |  |
| 2 | F | FCDIIb | 7.5 | 4 | FLAS, GTCS | O | 110 | II | Elisa, WB |  |
| 3 | M | FCDIIb | 6 | 3 | GES, GTCS | O | 60 | I | Elisa, WB |  |
| 4 | M | FCDIIb | 4 | 2 | FLAS, GTCS | P | 25 | I | Elisa, WB |  |
| 5 | F | FCDIIb | 10 | 5 | GTCS | P | 10 | I | Elisa, WB |  |
| 6 | F | FCDIIb | 5.5 | 3.5 | FLAS | Fr | 160 | III | Elisa, WB |  |
| 7 | M | FCDIIb | 8 | 7.5 | FLAS, GTCS | Fr | 20 | I | Elisa, WB |  |
| 8 | M | FCDIIb | 10.5 | 10 | FLAS, GTCS | T | 80 | IV | Elisa, WB |  |
| 9 | F | FCDIIb | 12 | 8 | FLAS, FBTCS | O | 45 | II | Elisa, WB |  |
| 10 | M | FCDIIb | 14 | 11 | GTCS | T | 20 | I | qPCR, IHC, WB |  |
| 11 | M | FCDIIb | 15 | 12 | FLAS, FBTCS | Fr | 75 | II | qPCR, IHC, WB |  |
| 12 | F | FCDIIb | 3.5 | 1.5 | GES | P | 130 | I | qPCR, IHC, IF |  |
| 13 | F | FCDIIb | 11 | 7.5 | GTCS | Fr | 140 | I | qPCR, IHC, IF |  |
| 14 | M | FCDIIb | 7 | 6 | FLAS, GTCS | Fr | 65 | II | qPCR, IHC, IF |  |
| 15 | F | FCDIIb | 4.5 | 2 | GES, GTCS | T | 130 | I | qPCR, IHC, WB |  |
| 16 | M | FCDIIb | 16 | 10 | FLAS, GTCS | P | 35 | II | qPCR, IHC, IF |  |
| 17 | F | FCDIIb | 5 | 3 | GES | Fr | 50 | I | qPCR, IHC, IF |  |
| 18 | F | FCDIIb | 6 | 3.5 | GTCS | O | 75 | I | qPCR, IHC, IF |  |
| 19 | M | FCDIIb | 7.2 | 5 | FLAS, GTCS | P | 20 | I | qPCR, IHC, IF |  |
| 20 | M | FCDIIb | 13 | 5 | GES | P | 65 | II | qPCR, IHC, IF |  |
| 21 | F | TSC | 2.5 | 1 | GES | P | 70 | I | qPCR, WB |  |
| 22 | F | TSC | 13 | 7.5 | GTCS | O | 25 | II | qPCR, IHC |  |
| 23 | F | TSC | 3 | 2 | GES, FBTCS | Fr | 60 | I | Elisa, WB |  |
| 24 | F | TSC | 17 | 8 | FLAS, GTCS | O | 150 | III | Elisa, WB |  |
| 25 | M | TSC | 16.5 | 7 | GTCS | Fr | 40 | I | Elisa, qPCR, WB |  |
| 26 | F | TSC | 6 | 5.5 | GES | T | 50 | I | Elisa, qPCR, WB |  |
| 27 | F | TSC | 7.5 | 3 | GTCS | O | 80 | I | Elisa, qPCR, WB |  |
| 28 | F | TSC | 16 | 6 | FLAS, GTCS | O | 100 | IV | Elisa, IHC, WB |  |
| 29 | M | TSC | 10.5 | 6 | GES | Fr | 120 | III | Elisa, IHC, WB |  |
| 30 | M | TSC | 14 | 9 | GES, GTCS | O | 40 | II | IHC, WB |  |
| 31 | F | TSC | 15 | 8 | PS, GTCS | T | 20 | I | Elisa, IHC, IF, WB |  |
| 32 | M | TSC | 16 | 8.5 | GTCS | P | 15 | III | Elisa, qPCR, IHC |  |
| 33 | F | TSC | 6 | 4 | FLAS, GTCS | P | 50 | I | qPCR, IHC, IF, WB |  |
| 34 | M | TSC | 8 | 4 | GES | P | 110 | I | qPCR, IHC, IF |  |
| 35 | M | TSC | 11.5 | 5 | GES, GTCS | T | 25 | II | qPCR, IHC, IF, WB |  |
| 36 | M | TSC | 7 | 5.5 | FLAS, GTCS | Fr | 20 | I | qPCR, IHC, IF |  |
| 37 | M | TSC | 12 | 6 | GTCS | T | 45 | I | qPCR, IHC, IF |  |
| 38 | F | TSC | 3 | 2.5 | GES | P | 75 | I | qPCR, IHC, IF |  |
| 39 | F | TSC | 10 | 8 | GES, GTCS | T | 25 | I | qPCR, IHC, IF |  |
| 40 | F | TSC | 5.5 | 4 | FLAS, GTCS | Fr | 30 | I | qPCR, IHC, IF |  |
| Abbreviations: F = female; M = male; FCD = focal cortical dysplasia; TSC = tuberous sclerosis complex; FIAS = focal impaired awareness seizure; GES = generalized epileptic spasm; GTCS = generalized tonic-clonic seizure; FBTCS = focal to bilateral tonic-clonic seizure; O = occipital lobe; T = temporal lobe; P = parietal lobe; Fr = frontal lobe; IHC = immunohistochemistry; IF = immunofluorescence; qPCR = real-time quantitative polymerase chain reaction; WB = Western blotting; Elisa = Enzyme Linked Immunosorbent Assay. | | | | | | | | | |  |
|  |  |  |  |  |  |  |  |  |  |  |

**Supplementary Table 3**

| Clinical features of control subjects | | | | | | |  |
| --- | --- | --- | --- | --- | --- | --- | --- |
| Case No. | Gender | Age  (year) | Cause of death | **PMI（h）** | Diagnosis | Seizure |  |
| 1 | F | 5 | Suffocation | 2 | Normal | None |  |
| 2 | F | 6.5 | Drowning | 4 | Normal | None |  |
| 3 | F | 7 | Suffocation | 5.5 | Normal | None |  |
| 4 | M | 20 | Motor vehicle accident | 3 | Normal | None |  |
| 5 | F | 8.5 | Drowning | 2 | Normal | None |  |
| 6 | F | 15 | Motor vehicle accident | 5 | Normal | None |  |
| 7 | M | 9 | Electric shock | 3.5 | Normal | None |  |
| 8 | F | 12.5 | Drowning | 4 | Normal | None |  |
| 9 | M | 16 | Electric shock | 2 | Normal | None |  |
| 10 | F | 8 | Motor vehicle accident | 5.5 | Normal | None |  |
| 11 | M | 17 | Drowning | 3 | Normal | None |  |
| 12 | M | 26 | Motor vehicle accident | 2.5 | Normal | None |  |
| Abbreviations: F, female; M, male; PMI, post-mortem interval (interval between death of a patient and removal of the brain before freezing or fixation). | | | | | | |  |
|  |  |  |  |  |  |  |  |
